# Farmers agronomic management responses to extreme drought and rice yields in Bihar, India

**DOI:** 10.1016/j.agwat.2025.109830

**Published:** 2025-11-01

**Authors:** Maxwell Mkondiwa, Avinash Kishore, Prakashan Chellattan Veetil, Sonam Sherpa, Satyam Saxena, Bhavani Pinjarla, Anton Urfels, Shishpal Poonia, Anurag Ajay, Peter Craufurd, Ram Malik, Andrew McDonald

**Affiliations:** a International Maize and Wheat Improvement Center (CIMMYT), New Delhi, India; b International Food Policy Research Institute (IFPRI), New Delhi, India; c International Rice Research Institute (IRRI), New Delhi, India; d International Maize and Wheat Improvement Center (CIMMYT), Kathmandu, Nepal; e Technology Implementation Cell-PMFBY, Sustainable Inclusive Growth, UNDP, New Delhi, India; f International Rice Research Institute (IRRI), Los Banos, Philippines; g Cornell University, USA; h Water Resources Management Group, Wageningen University, Wageningen, Netherlands

**Keywords:** Tubewell irrigation, Machine learning, Late monsoon onset, Crop abandonment

## Abstract

In 2022, the Indian state of Bihar experienced its sixth driest year in over a century. To document the consequences and farmer responses to the meteorological drought, real-time survey data was collected across 11 districts of Bihar. We then developed a causal machine learning model to quantify drought impacts on rice production and to characterize how access to affordable irrigation from electric pumps mitigated productivity losses. This model addresses the empirical challenge of conducting a counterfactual causal analysis when a factor like drought affects nearly all sampled farmers. In the 2022 event, drought led to rice acreage reduction, transplanting delays, damage to seedling nurseries, and higher use rates of supplemental irrigation. For fields that were planted, average yield losses from water stress were estimated as 0.94 t/ha (∼23 % yield loss) with these losses reduced by 0.3 t/ha in fields with access to electric tubewells. Agronomic management practices such as earlier transplanting were also identified as complementary strategies that increased the adaptation value of investments in irrigation. To reduce the impact of drought in Bihar, additional investments in electric irrigation infrastructure are needed along with focused extension efforts and decision support systems that empower farmers to make economically and sustainably rational use of available water resources to maintain yield and profitability.

## Highlights

• Affordable irrigation is considered a panacea for recurrent droughts but not much known for extreme droughts.
• In 2022, Bihar experienced a sixth driest agricultural season.
• Causal machine learning framework allowed testing of drought impact on crop management and yields.
• Affordable irrigation requires complementary agronomic services to deal with extreme droughts.

## Graphical Abstract

**Figure:**
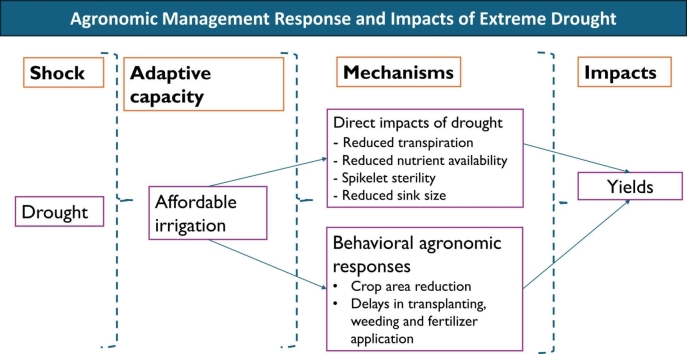

## 1 Introduction

The increasing frequency of droughts due to climate change presents enormous challenges to the livelihoods of millions of farmers in developing countries, with impacts ranging from farm abandonment to reductions in cropped acreage and lower yields for fields that are cultivated (see Bordner et al., 2015). For irrigated lowland rice systems, water limitations are increasingly recognized as core production constraints that strongly contribute to yields gaps even under typical growing conditions (Nayak et al., 2024, Haefele et al., 2016). Nevertheless, from the broader perspective of long-term agricultural development, Birthal et al. (2015) and Birthal et al. (2021) note that although the frequency of meteorological drought has increased in India, the impacts to rice yields have weakened due to expansion of irrigation facilities and increased availability of improved varieties, demonstrating the importance of adaptive capacity for stabilizing agricultural productivity in a changing climate.

In 2022, the Indian state of Bihar experienced its sixth driest year in over a century. From June to September in 2022, rainfall during the South-West monsoon period in Bihar was 31 % below the normal. The Indian Meteorological Department (IMD) reported significant rainfall deficits in 34 of the 38 districts. The most rainfall-deficient districts in the state were Bhagalpur (-59 %), Lakhisarai (-54 %), Sitamarhi (-53 %), Sheikhpura and Saran (-50 %, each) and Saharsa and Katihar (-48 %, each).^1^ In response to the drought, the Government of Bihar provided diesel fuel and electricity subsidies to some farmers to support irrigation.^2^ Irrigation in Bihar is principally derived from shallow groundwater, with pumps powered by either diesel or electricity (Ajay et al., 2022).

Government programs to supply affordable energy to support irrigation in times of drought are common in South Asia, but evidence of impact from these strategies is limited. Nevertheless, as is common in South Asia, studies by Urfels et al. (2020) in Terai region of Nepal and Kishore (2019) in Bihar State of India have reported that electric pumping is much less costly than diesel pumping and that this favours increased irrigation intensity (i.e., number of irrigation events per season). Specifically, Srivastava et al. (2021) report that the cost of groundwater extraction by shallow tubewells in Bihar was INR 2.4/m^3^ for diesel pumps and INR 0.6/m^3^ for electric tubewells (1 USD=80 INR).

Adapting to drought, however, is a complex challenge that is mediated by farmers perceptions, awareness and appetite for risk, as well as behavioural responses that impact agronomic management and yield outcomes beyond irrigation. For example, farmers may be less willing to invest in critical inputs such a fertilizers if the production year is deemed too risky (Cooper et al., 2008). There are also methodological challenges for understanding complex responses to drought. Farmer behaviour is heterogenous and is informed by differences across diverse set of factors such as land type, level of market integration, and access to information from extension services. Heterogeneity of subgroups poses a major methodological challenge because sample splitting is not possible for all possible combinations of factors that likely shape responses. Recent studies have suggested using causal and double machine learning approaches to analyse the impacts of weather and other shocks on farming outcomes. For example, Stetter and Sauer (2021) used a generalized random forest model to analyse the complex relationships between weather, farm management, biophysical conditions, and total factor productivity. Vilgalys (2023) compared standard machine learning models (e.g., LASSO), panel data models and debiased double machine learning in analysing climate adaptation. Debiased double machine learning—in which a drought exposure probability model is used in the first stage then predicted probabilities are used to inversely weight the second stage non-parametric robust score estimates of drought impact on yields, was identified as the best approach. In this study, we use this causal random forest model and debiased/double machine learning (DML) approach (Athey et al., 2019, Semenova and Chernozhukov, 2021) to analyze the impact of drought on crop management practices, including irrigation with and without the benefits of energy subsidies, and yield outcomes.

With respect to causal attribution,^3^ the key concern in measuring drought impact is the potential measurement error of the self-reported, and satellite data derived measures of drought (e.g., standardized precipitation index (SPI), and deviation of normalized vegetation index (DNDVI)). These measures are commonly used to assess the idiosyncratic drought shocks that may not be easily identified from generalized weather station data. In this paper, we prioritize the use of subjective (farmer reported) measures of a drought because our concern is with farmer behavioural responses. Another empirical challenge for causal identification of extreme drought impacts is due the adaptation options in terms of within season adjustments in input use and farm management decisions (e.g., time of transplanting). Farmers with access to electric tube wells will likely have other characteristics that we cannot control for in the model including access to credit and liquidity. This can be resolved in the causal random forest framework using the modified causal forest approach that allows estimation of causal moderation effects.^4^ To evaluate the causal impact of weather extremes on agricultural outcomes, it is common to use panel data (e.g., Carter et al., 2018, Hsiang, 2016, Li, 2023, Merel and Gammans, 2021). We do not have panel data as the goal our study was to assess a drought in real-time. To deal with this data challenge, it is standard practice in the Ricardian climate change econometrics literature (for cross-sectional data) and in this study to either control for or interact the adaptation options with the climate variables (Su and Chen, 2022, Bekele et al., 2024).

The main objective of this paper is to examine the heterogenous impacts of drought exposure on crop management decisions and rice yields as influenced by access to inexpensive irrigation. Behavioural responses to drought characterized in this paper include shifts cropped area, timing of crop establishment, weed management, irrigation management and nutrient management. We improve on standard methods for cross-sectional analysis (interaction and controls) by using a causal random forest model that allows us to estimate the causal effect of drought and associations to adaptation strategies while interacting all the necessary variables.

## 2 Materials and methods

### 2.1 Conceptual framework

**Fig. 1:**
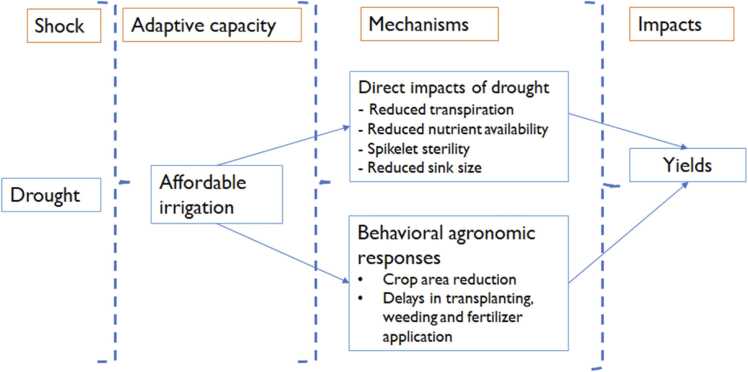
Drought effects and behavioural agronomic responses (source: adapted from Haefele et al., 2016; Jain et al., 2015; Cui, 2020).

Drought stress in rice production is manifested in several ways. According to Haefele et al. (2016), there are direct and indirect effects of a drought (see Fig. 1). We follow this structure to investigate the effects of drought on changes in management practices and how some of the farmer endowments like electric tube wells help in mitigating the drought effects. There are three direct pathways. First, drought directly reduces transpiration which limit growth and biomass development. Second, it reduces the availability and uptake of nutrients by plants, and photosynthesis by plants. Lastly, a drought induces spikelet sterility and reduces sink size.

These biophysical effects are evidenced in reduced grain size and grain quality. A drought also affects agronomic management through time management disruptions especially through delayed planting. These effects may be mitigated and adapted by improving access to irrigation, changes in system efficiency by management practices and through changes in genotypes. The indirect effects are due to decision-making lapses and changes including low input use and adoption of stress tolerant varieties some of which may be low yielding.

We characterize several crop management responses to droughts and assess how access to cheaper and efficient irrigation through electric tube wells influences such responses. The crop management practices include crop land use, timely transplanting, seedling age at transplanting, weed management and nutrient management. On crop land use, we hypothesize that in the face of a drought, some farmers may decide not to grow rice and leave the land fallow. For crop establishment, we hypothesize that early kharif (June and July) season drought results in delays in transplanting (as stated by Balwinder-Singh et al., 2019), use of old seedings, and wastage of nursery. This is the case because most farmers wait until the onset of monsoon rains to prepare nurseries and heavier monsoon rains to transplant (McDonald et al., 2022). For irrigation management, we conjecture that a drought results in increase in number and average number of hours for each irrigation. For weed management, we hypothesize that weeds will outcompete rice crop such that farmers will need more manual weedings and herbicide sprays. On nutrient management, we hypothesize that the farmer would respond to drought by overapplying more fertilizers than normal as they attempt to remedy the poor crop appearance or underapplying with the anticipation of poor crop performance or a chance of failure. In addition to these, the historical occurrences of drought and other shocks may also affect the behavioural responses in that farmers in risk prone regions will not invest much in good seed or fertilizer due to high risk of losses (Haefele et al., 2016). Jain et al. (2015) also suggests that decisions to alter cropping strategies may also be affected by biophysical (e.g., soil type), economic (e.g., assets), social (e.g., networks) and cognitive (e.g., risk perceptions) factors. The adapted conceptual model therefore points to the need to consider multiple pathways (including irrigation, genetics, and crop management) when estimating the causal impact of droughts. Our conceptual model is consistent with the directed acyclic graph (DAG) showing how droughts affect yields as proposed by Li (2023).

### 2.2 Empirical model

#### 2.2.1 Naïve estimating equations

We consider two “naïve” baseline estimating equations normally used to analyze impacts of extreme weather events on agriculture in a cross-sectional data context. These can be referred to as “interaction” model (as in Zaveri and Lobell, 2019) and “mediating effects” model. Our baseline estimating Eq. (1) for interactions model estimates the relationship between drought exposure and cheap irrigation availability on crop management practices.

(1) $$Y=\beta_{0}+\beta_{1}\mathrm{Drought}+\beta_{2}\mathrm{Pump Type}+\beta_{2}\times \mathrm{Drought}\times \mathrm{Pump Type}+\eta Z+\epsilon$$

Where Y are outcome variables area under rice, time to transplanting, and age of seedlings. Z is a set of control variables including source of irrigation (e.g., canal, tube well, dug well), elevation, soil quality variables, soil texture (sandy), and total land holding acreage. In another baseline model, the mediating effects model, we do not include the interaction model.

These models focus on the cross-sectional variation in exposure and impacts. However, this may be misleading because of endogenous exposure. That is, the locations that had a drought may also be predominantly drought locations from a historical perspective which affects the crop management practices that are followed. To deal with this challenge, we include specifications in which the exposure and outcome variables are first differences from 2021.^5^ This is done retrospectively by responses of the farmers regarding the crop management practice changes between a normal year and a drought year.

(2) $$Y_{t}-Y_{t-1}=\beta_{0}+\beta_{1}\mathrm{Drought}+\beta_{2}\mathrm{Pump Type}+\beta_{1}\times \mathrm{Drought}\times \mathrm{Pump Type}+\eta Z+\epsilon$$

In the case of farmer reported drought, we collected data only for the 2022 monsoon season while for the appropriate satellite derived measures we also collected for the 2021 monsoon season.

These estimating equations are problematic for several reasons. Firstly, one requires a credible control group to estimate the causal impact of drought and access to irrigation. This is problematic in the case of droughts because all farmers within a district may have been affected by the drought and in most cases either most of the farmers are affected or not affected.^6^ The variation is therefore dependent on few observations if district fixed effects are used and if district fixed effects are not used the cross-sectional counterfactual is dependent on farmers who have substantially different agricultural conditions.^7^ The causal random forest approach we present next addresses these concerns by allowing many covariates thereby ensuring that even without district fixed effects the comparisons are across similar farms.

#### 2.2.2 Causal machine learning framework

According to Kurukulasuriya et al. (2011), the ideal approach of estimating the impacts of irrigation on yields under droughts in a cross-sectional analysis is to estimate the probability of making an irrigation decision and then its effects on the outcomes. Alternatively, we can estimate a drought exposure model using drought experience for the individual farmer as outcome variable and geographical variables as controls. This can be estimated using limited dependent variable estimators like logit and probit or using machine learning estimators like probability random forest. Then one calculates propensity scores that are then used as weights for the model. This procedure is called the inverse probability weighted (IPW) estimation approach. This represents a conditional average treatment effect estimator rather the mediation or interaction estimators in the conventional estimating equations.

In this paper, we use a causal random forest variant of this estimator following Athey et al. (2019)^8^ so as to generate credible control group and estimate heterogenous treatment effects across geographical gradients and crop management decisions. This estimator also involves two stages: the drought exposure model and drought impact model. The estimating equation for the exposure model is:

(3) $$D=\alpha_{0}+\alpha_{1}Z+\mu \;$$

Where D is 1 if the farmer experienced a drought, 0 otherwise. Z is a vector of control variables including location dummies (e.g., district), canal accessibility, population density, soil sand, soil carbon, and elevation.^9^ We use probability random forest model to compute the predicted probability scores.

In the drought impact model, we use the computed propensity scores as weights in an augmented inverse probability weighted estimator (AIPW). The causal random forest model is estimated with drought exposure variables, access to affordable irrigation and other agronomic management practices (for the yield model only) as independent variables. As outcome variables, we use yields and several agronomic practice outcome variables including area planted, weeding expenditures, transplanting dates, and age of seedlings. The conditional average treatment effect (CATE), i.e., yield loss due to drought, can be represented as (Athey et al., 2019, Schmidt et al., 2025)

(4) $$\tau \left(x\right)=\left[Y_{i}\left(1\right)-Y_{i}\left(0\right)|,X_{i}=x\right]\;$$

Where Y(1) is outcome (e.g., yield) with drought and Y(0) is outcome (e.g., yield) without drought. X is a matrix of control variables (which includes: elevation, number of irrigations, canal irrigation dummy, rice variety, transplanting day, age of rice nursery, soil organic carbon, soil sand, population density) and moderator variables of interest (own electric pumps). These conditional average treatment effects (CATE) represent the impacts of drought exposure on the different outcomes.

Following the potential outcomes framework, two key assumptions of unconfoundedness (drought incidence is as good as random conditional on control variables) and overlap (common support) should be satisfied for causal treatment estimation. Given that farmers are not randomly assigned to a drought, this assumption is likely violated when using observational data as we do. We however assumed a selection on observables design (while acknowledging that this may not be enough to address endogeneity concerns) by including all confounders to a farmer reported drought event as control variables including soil texture, elevation, and access to canal irrigation. Similarly, farmers located in drought areas may be fundamentally different from those in other areas thereby violating the overlap assumption when using observational data. These concerns are addressed in the causal random forest approach in that the first stage involves fitting a probability random forest that is used to weight (only observations with common support are retained and used for the average treatment effects) the second stage estimates.

To examine if having electric tube wells helped the farmers to adapt to the drought better, we classify the estimated CATE by pump type or use double debiased machine learning (DML) estimator to analyse the impact of access to electric tube wells on the drought effect. We also examine the differential drought effects across transplanting dates, number of irrigations and biophysical factors like soils and elevation. To ensure that our causal machine learning are robust, we conducted calibration tests using *test_calibration* function in *grf* package to ensure that mean forest prediction and differential forest prediction are all correct.

### 2.3 Data

To understand the agronomic management practices in the face of a drought, we conducted household farmer surveys during the 2022 drought, one of the most challenging droughts in Bihar. We interviewed 520 randomly selected farmers from October to December 2022. We focused the questions on the on the agronomic management decisions in the monsoon (rice) season from June to November 2022. In this survey, we couldn’t collect yield data as some of the farmers had not yet harvested. In June 2023, we therefore conducted a follow-up telephone survey in which we attempted to call up all farmers who were interviewed in 2022. We managed to interview 339 of the initial 520 farmers. To validate the responses in the telephone survey, we followed up with another call for which only 219 farmers responded. In this paper, we use data from about 518 farmers that grew rice of which only 512 responded on the question of interest regarding drought experience in 2022. Table 1, Table 2 shows the distribution of the number of sampled farmers by district and drought experience. Droughts were experienced by 344 farmers (67 %) residing in 9 of the 11 districts. In 7 of the districts (Banka, Gaya, Lakhisarai, Madhepura, Samatipur, Vaishali and West Champaran), all farmers within the district reported that they experienced the drought. None of the farmers in Arah and Rohtas reported of experiencing a drought.

**Table 1:** Number of farmers interviewed in the drought impact survey, Bihar 2022.

	Did you experience impact of drought in 2022?		
District	No	Yes	Total
Arah	56	0	56
Banka	0	38	38
Buxar	65	22	87
East Champaran	6	50	56
Gaya	0	39	39
Lakhisarai	0	40	40
Madhepura	0	41	41
Rohtas	41	0	41
Samastipur	0	45	45
Vaishali	0	42	42
West Champaran	0	27	27
**Total**	**168**	**344**	**512**

Note: 512 is the total sample for those who provided credible responses to the survey questions.

**Table 2:** Satellite derived data and farmer reported measures of drought.

		Farmer reported drought impact, 2022		
Meteorological drought (SPI<1)	Drought incidence	No drought (n = 164)	Drought (N = 340)	Total
JJAS	No drought	124	202	326
	Drought 2022 only	40	138	178
JJ	No drought	60	186	246
	Drought 2022 only	104	154	258
Agricultural drought (DNDVI<0)				
JJAS	No drought	137	231	368
	Drought 2021 only	2	30	32
	Drought 2022 only	29	68	97
	Drought both 2021 and 2022	0	10	10
JJ	No drought	64	162	226
	Drought 2021 only	6	53	59
	Drought 2022 only	87	91	178
	Drought both 2021 and 2022	11	33	44

Note: JJAS=June, July, August, and September; JJ=June and July.

**Fig. 2:**
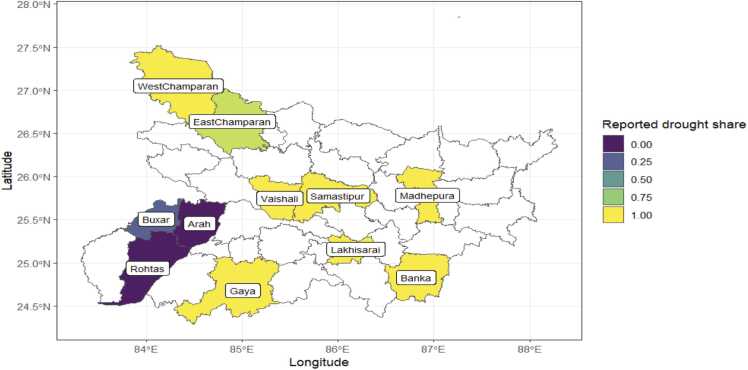
Sampled districts and reported drought proportion in Bihar.

Fig. 2 plots the spatial distribution of farmers who reported to have faced drought. There is spatial pattern with only few sampled districts (Arah and Rohtas) in the southwestern part of Bihar not reporting droughts. Coincidentally, these are also canal irrigation areas. This spatial pattern of droughts is problematic for causal identification of the impacts of drought on rice yields because the yield differences may be due to other geographical factors.

**Fig. 3:**
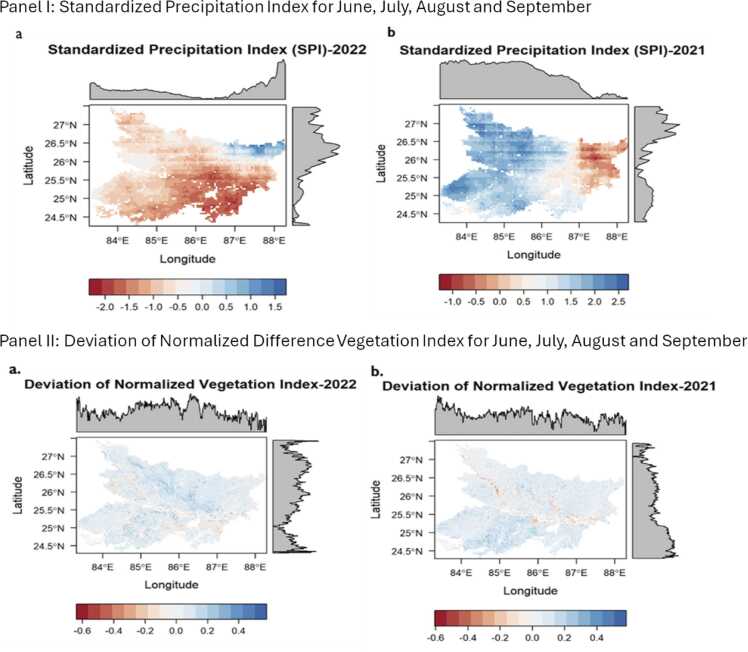
Meteorological and agricultural drought in Bihar, 2021–2022. Note: Panel I(a) shows the standardized precipitation index for 2022 (extreme drought year) for monsoon months of June, July, August and September while panel I (b) shows standardized precipitation index for the corresponding months in 2021. Panel II(a) shows the deviation of normalized difference vegetation index for 2022 (extreme drought) while Panel II (b) shows the deviation of normalized difference vegetation index for 2021.

To validate farmer’s reporting of droughts, we use gridded datasets on precipitation from the CHIRPS to compute two measures of drought: Standardized Precipitation Index (SPI)^10^ and Deviation of Normalized-Difference Vegetation Index (DNDVI). These refer to meteorological drought and agricultural drought respectively. Fig. 3 shows the spatial patterns of meteorological drought and agricultural drought for 2021 and 2022. The spatial patterns in the reported 2022 droughts and computed indicators are consistent.

We follow the World Meteorological Organization in defining SPI estimates less than −1 as drought. For Deviation of Normalized Vegetation Index, we use DNDVI< 0 as drought.^11^ We find that in aggregate, there are disparities in farmer reported and the satellite data derived indicators. We use the farmer reported measures of drought in the paper because the paper is focused on behavioural responses to the perceived drought.

## 3 Results

### 3.1 Descriptive statistics

**Fig. 4:**
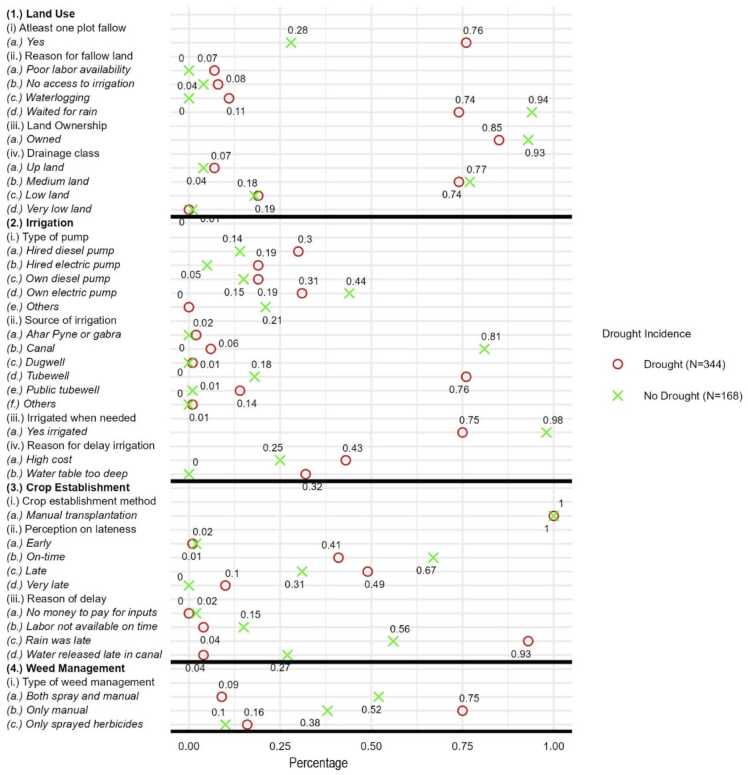
Summary statistics of key variables by reported drought incidence, Bihar 2022.

Fig. 4 reports the summary statistics for key variables by reported drought incidence. The rest of the descriptive statistics are reported in Table A1. On land use, farmers reduced rice acreage and left some plots to fallow. About 60 % of the farmers reported that they left at least one of their plots to fallow. The main reasons for keeping some of the plots fallow included that they were waiting for rain (74 %), and the plot was water logged (10 %). In terms of land ownership, most of the farmers we interviewed own their land (85 %) and the land is in medium drainage class (74 %).

On irrigation, the farmers who own electric pumps are about 37 %, with the proportion higher for those who did not experience drought (44 %) as compared to those who experienced drought (31 %). Only 75 % of the farmers were able to irrigate when they needed to for those who reported a drought as compared to 98 % for those who did not experience a drought. The two main reasons for the delay in irrigation were the high cost of irrigation and that the water table was too deep. This implies that physical water resource constraint is important beyond cost and awareness of proper scheduling of irrigation.

All farmers except one established their rice by transplanting. The median transplanting date was by 20th July. According to McDonald et al. (2022), the optimal transplanting date is 13th July for long duration varieties and by 3rd August for medium duration varieties. For the drought year, 40 % of the farmers perceive that they were late with transplanting with 48 % of those reporting a drought stating they were late as compared to 31 % for those not reporting a drought. The average age of nursery before transplanting was about 30 days. For those who were late, the main reason was that they were waiting for the rains— 93 % for those who experienced drought and 56 % for those who did not.

On weed management, farmers who did not experience drought used both manual weeding and herbicides for weed management (52 %) while the majority of those who experienced drought used manual weeding only (75 %). Farmers in Ara and Rohtas districts did not experience drought because these are shallow low land and there is some canal irrigation. The average paddy yield in these districts is more than other districts under reference. With higher yields weed flora becomes simple and herbicides are more successful and more economical. Farmers who experienced drought, weeds outcompeted crop and weed flora becomes more complex. Herbicides are less successful in that scenario. That is why there is less use of herbicides. Manual weeding especially for complex flora is very costly.

**Fig. 5:**
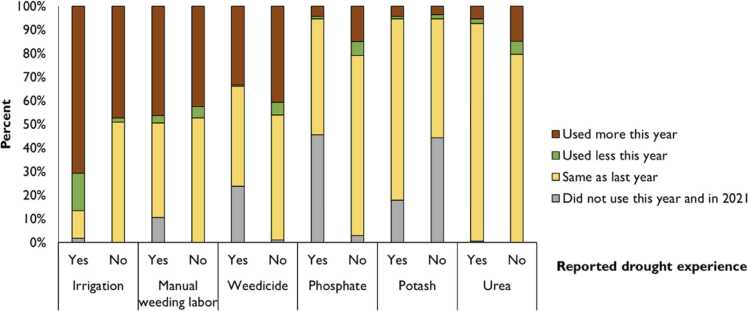
Changes in irrigation, weed management and fertilizer application by drought experience.

The concern with the cross-sectional comparisons in Fig. 4 is that the observed differences may not be attributed to the drought but that these locations are historically different on those factors. To address this concern, we asked generic perception questions to farmers on whether they have changed any of the crop management decisions in 2022 as compared to 2021. Fig. 5 shows the crop management practice changes between 2022 and 2021. Much of the literature suggests that droughts lead to overapplication of irrigation, herbicides, and fertilizers (see Caton et al., 2010). On irrigation, about 70 % of farmers in drought areas irrigated more in 2022 than in 2021 as compared to about 50 % for those who did not experience a drought. Herbicides are expected to be over applied in a drought season because there is increase of C4 weeds and that the rice crop will fail to outgrow the weeds some of which emerge if the crop canopy is not well established (Caton et al., 2010, Ramesh et al., 2017). The results however show a contrary view in which the percent of farmers who increased use of herbicides is not different for those who faced drought as compared to those who did not face drought. Similarly, farmers did not change any of the nutrient management practices. In the foregoing analyses, we limit our analyses to land use, crop establishment, irrigation and weed management leaving aside nutrient management.

Using the sample of farmers who were able to respond to the telephone survey and provided consistent responses across the surveys (about 300), we analyzed the yields by drought experience and districts. Table 3 shows the summary statistics of drought occurrence and rice yields across districts. This table demonstrates the challenges one would face to estimate the impacts of drought on yields. Except in Buxur and East Champaran, all the farmers either experienced or did not experience drought in the other districts. This means that the comparisons can be affected by the yield levels in each of the districts.

**Table 3:** Drought exposure and rice yields in Bihar, 2022.

District	Faced drought	N obs	Mean (kg/ha)	SD
Arah	No	35	4957.14	1139.58
	Yes	0		
Banka	No	0		
	Yes	23	3344.89	1398.07
Buxar	No	40	4831.58	1345.92
	Yes	16	5294.19	927.20
East Champaran	No	5	3395.52	1282.81
	Yes	36	2679.38	1000.87
Gaya	No	0		
	Yes	31	4176.96	1673.12
Lakhisarai	No	0		
	Yes	23	4035.24	1569.95
Madhepura	No	0		
	Yes	20	4151.52	1764.95
Rohtas	No	31	5621.01	1393.30
	Yes	0		
Samastipur	No	0		
	Yes	24	4287.09	1328.24
Vaishali	No	0		
	Yes	28	4042.09	1555.02
West Champaran	No	0		
	Yes	23	3098.86	1725.76

### 3.2 Drought and crop land use

**Fig. 6:**
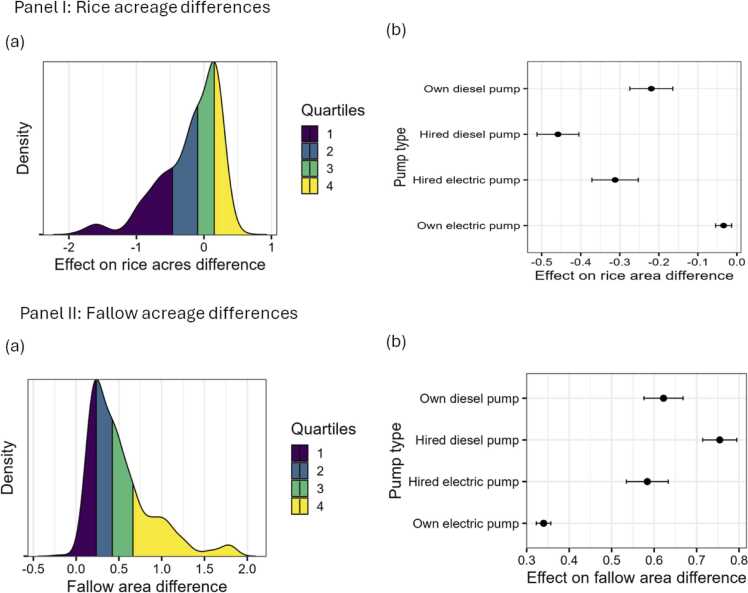
Distribution of conditional average treatment effects of farmer reported drought on rice and fallow acreage. Note: Panel I(a) shows the rice acreage differences between those who faced drought and those who did not while panel I. Similar density plot with density values is in appendix B. Panel I (b) differentiates these differences by pump type. Panel II(a) shows the fallow acreage differences between those who faced drought and those who did not while panel II (b) differentiates these differences by pump type.

Farmers responded to the drought by leaving some land fallow and reducing area allocated to rice in 2022 as compared to 2021. Fig. 6 panel I shows the distribution of conditional average treatment effects of drought on the difference between acreage of rice in 2021 and 2022. For over 75 % of the farmers, drought led to a reduction in crop acreage. The average treatment effect of drought was 0.23 acres reduction in rice acreage as compared to the previous year. Much of this reduction is for those who used hired diesel or electric pumps. These pumps are both expensive and difficult to arrange as such farmers may have given up on hiring when they experienced the drought. Owning electric tubewell effectively abates this effect.

### 3.3 Drought and crop establishment

**Fig. 7:**
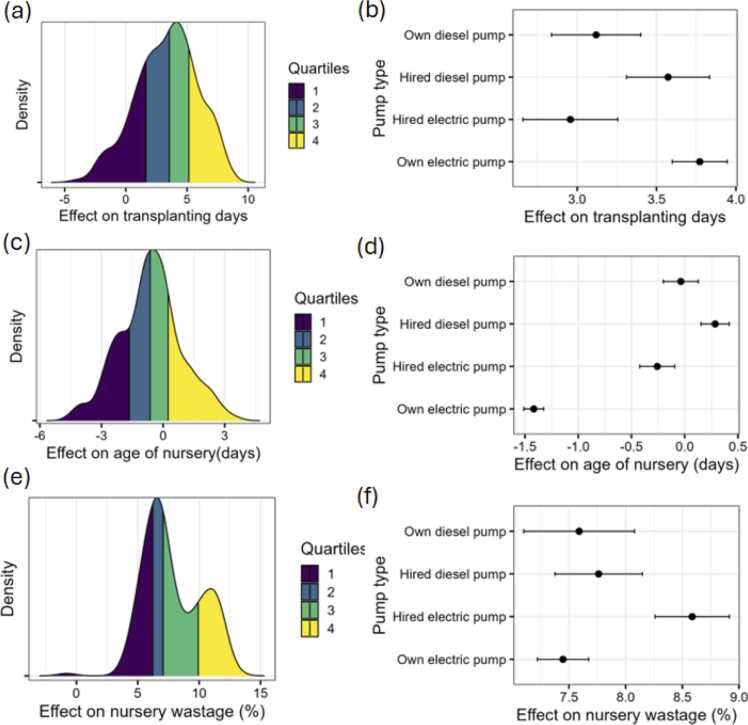
Transplanting days, age of nursery and nursery wastage. Note: Panel (a) shows the transplanting days differences between those who faced drought and those who did not while panel (b) differentiates these differences by pump type. Panel (c) shows the age of nursery differences between those who faced drought and those who did not while panel (d) differentiates these differences by pump type. Panel (e) shows the percentage difference in nursery wastage between those who faced drought and those who did not while panel (f) differentiates these differences by pump type.

Fig. 7a shows the impact of drought on delays in transplanting. Those who experienced droughts delayed transplanting by about 2.8 days on average. Surprisingly, the delays were more for those who have access to own electric pumps (Fig. 7b). To understand the reasons for this behavioural response, we explore the decision in relation to other decisions on nursery age and wastage. We find that farmers who reported a drought did not differ on average from those who didn’t face a drought on the age of their rice nurseries (Fig. 7c). However, those who had own electric pumps were able to transplant younger seedlings than those using expensive forms of irrigation (Fig. 7d). Additionally, at the extensive margin, 39.3 % of the farmers reported to have some of the nursery wasted. About 87 % of these farmers are those who have experienced drought. On the intensive margin, the effect of drought on the percent of nursery wastage was about 7 percentage points (Fig. 7e). These were not different for those owning electric pump and hiring or owning diesel pump. However, the amount of wastage was higher for those hiring electric pumps (Fig. 7f).

### 3.4 Drought and irrigation management

**Fig. 8:**
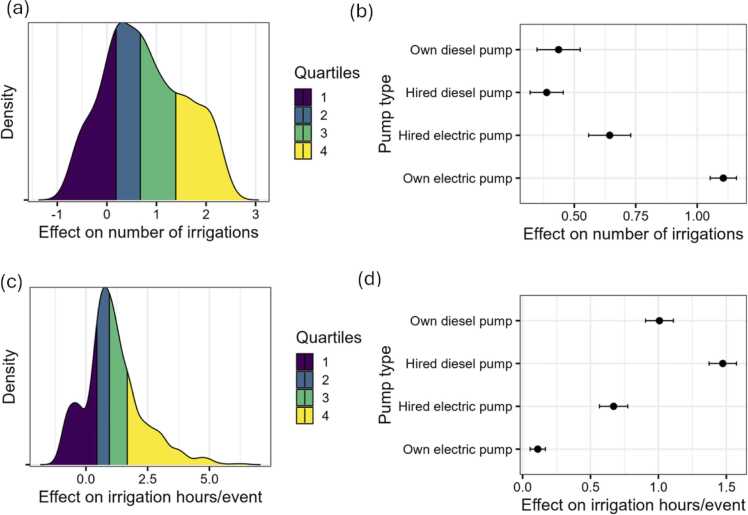
Number of irrigations and irrigation hours per event. Note: Panel (a) shows the number of irrigation differences between those who faced drought and those who did not while panel (b) differentiates these differences by pump type. Panel (c) shows the irrigations per irrigation differences between those who faced drought and those who did not while panel (d) differentiates these differences by pump type.

In irrigated cropping systems, droughts inevitably prompt the farmer to apply more irrigation especially if the irrigation cost is affordable. Fig. 8a shows that drought indeed resulted in additional irrigation events with those with access to own electric pumps applying a full additional irrigation while those who own or hire diesel pumps applying less than 0.5 additional irrigation when faced with a drought (Fig. 8b). However, in terms of increase in the irrigations hours per event (Fig. 8c), those who use own electric pump use less hours (Fig. 8d). This is the case because electric tubewells have bigger pumps and are more efficient at dispersing a higher volume of water per unit of time.

### 3.5 Drought and weed management

**Fig. 9:**
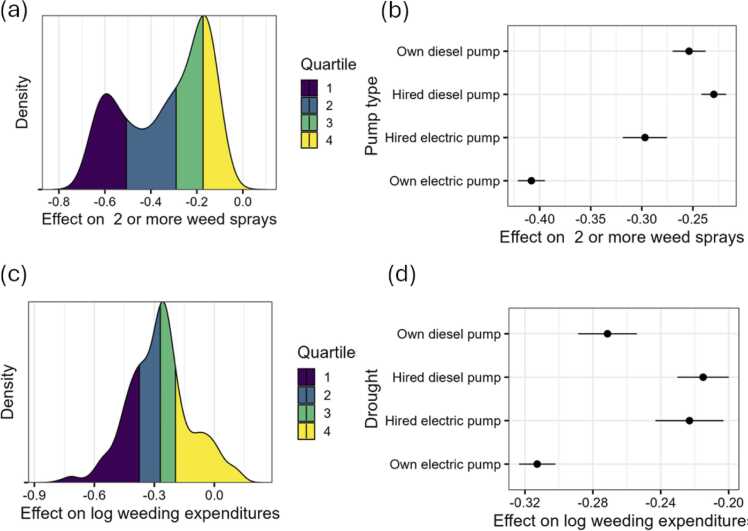
Drought impact on weed sprays and log weeding expenditures. Note: Panel I(a) shows the weeding incidence (two or more weedings) differences between those who faced drought and those who did not while panel I (b) differentiates these differences by pump type. Panel II(a) shows the logarithm weeding expenditure differences between those who faced drought and those who did not while panel II (b) differentiates these differences by pump type.

Fig. 9 part I shows the distribution on the effect of drought on 2 or more weed sprays. Surprisingly, those who face a drought are spraying herbicides less. This is contrary to the hypothesis that droughts result in weed flush which increases the incidence of multiple sprays. However, the reduction in the acreage to rice may also mean that parts of the field prone to early growth of weeds were not planted with rice. The complexity of weed species distribution and intra-seasonal growth dynamics make it difficult to conclude the direction of the drought effects. Those with own electric pumps are however spraying less than those who own or hire diesel pumps. This may also be the effect that increased and timely irrigation in drought affected farmers is effective at reducing weed growth. Irrigation itself helps creating competition in favor of the crop. This pattern is also reflected in the weeding expenses (Fig. 9 part II).

### 3.6 Drought impact on yields and adaptation pathways

**Fig. 10:**
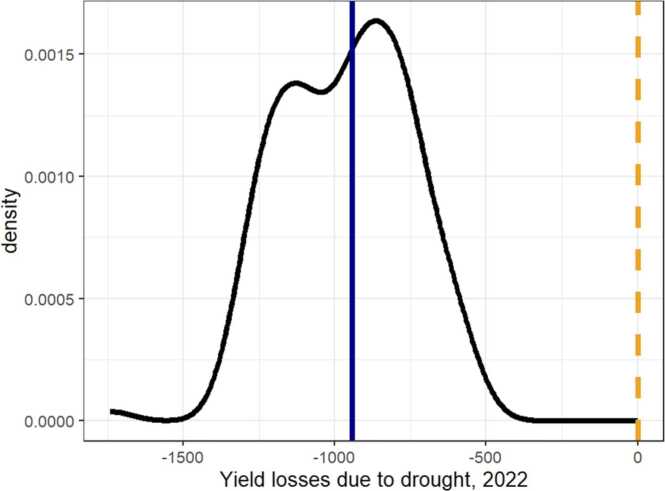
Yield losses due to drought, 2020. Note: The blue solid line represents the average treatment effect at −942 kg/ha.

Causal analysis of the impacts of droughts on crop yields requires a credible control group. In the case of our study, this is problematic because the drought either occurred or did not occur to all farmers within a district except for two districts. We addressed this challenge by using a causal random forest modelling framework which bases the control not only on the basis of not experiencing a drought but also on the basis of biophysical and farm management characteristics (e.g., elevation, number of irrigations) instead of district fixed effects.^12^ Fig. 10 reports the distribution of the 2022 drought impact on rice yields. There is enormous heterogeneity in the drought losses ranging from about 0.5t/ha to 1.5t/ha. On average, farmers who planted rice in a drought affected area lost about 0.94 t/ha (about 23 %) solely due to the drought.

**Fig. 11:**
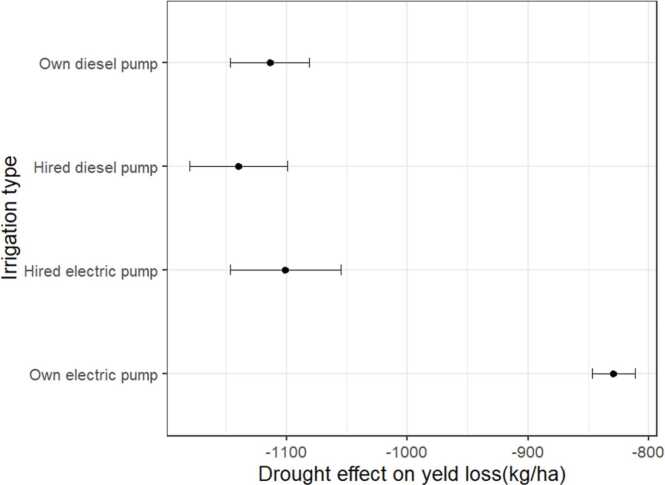
Effect of irrigation type on yield losses too drought.

**Figure A2:**
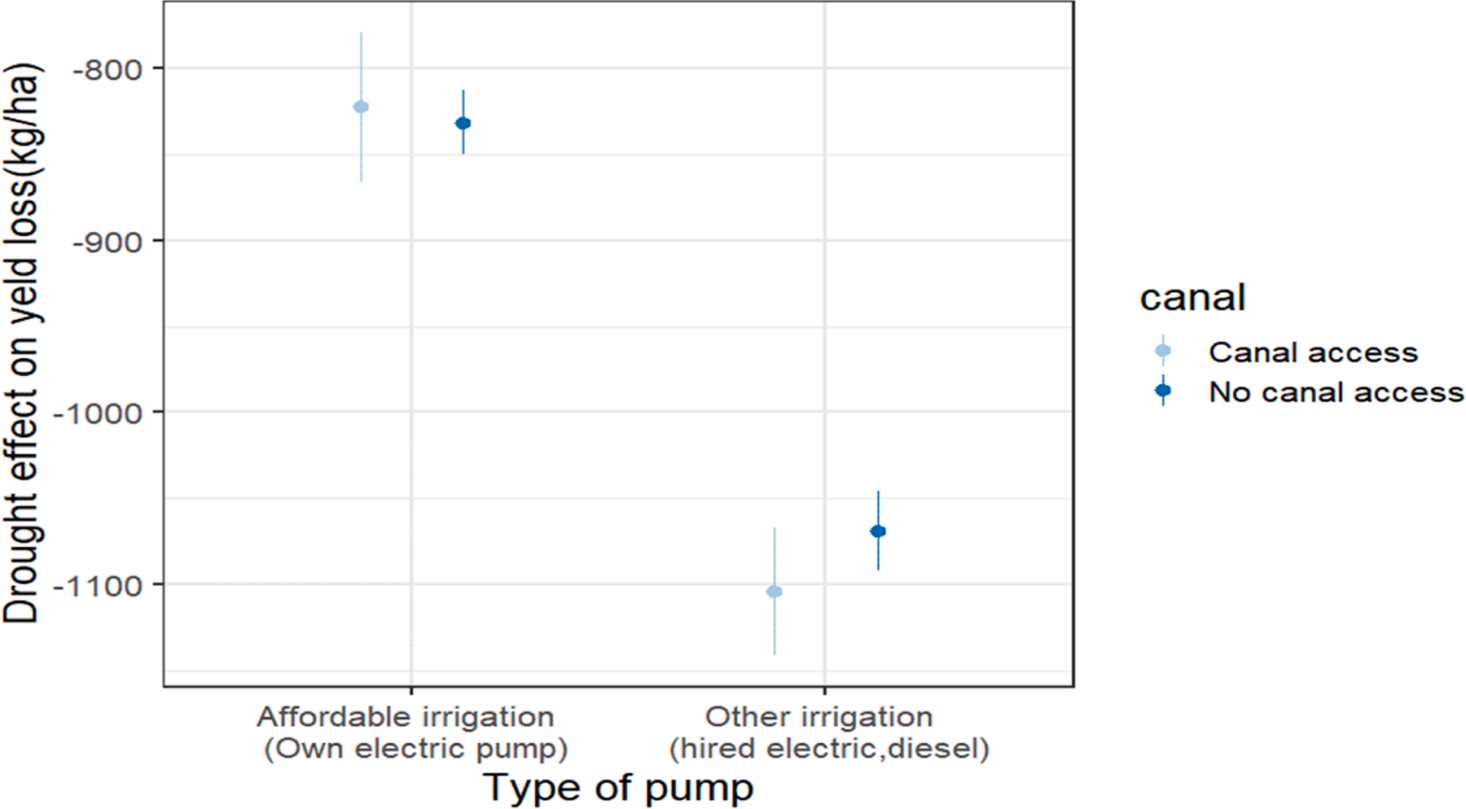
Drought impact on yield losses by pump type and access to canal

**Figure A3:**
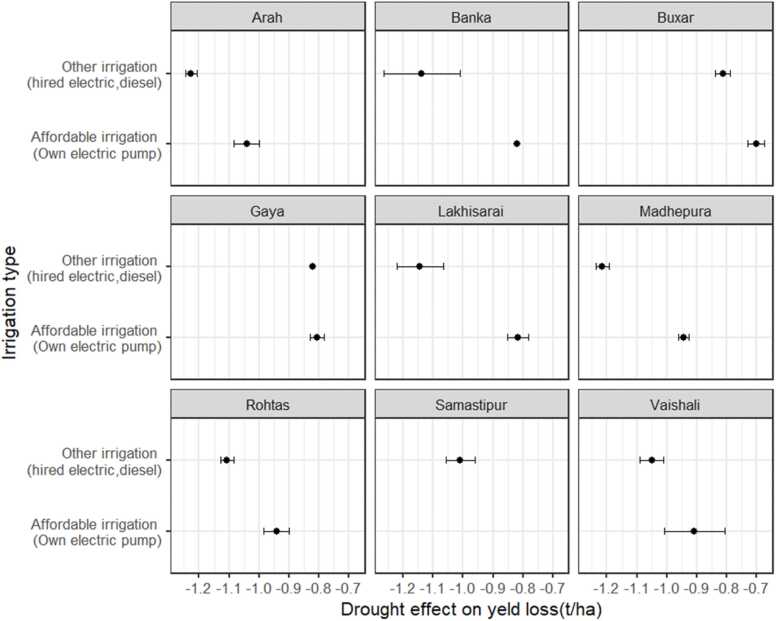
Drought effect on yield losses by irrigation type and districts

Given these individual level estimates of the drought losses, we can check who loses less depending on the type of irrigation pump. We find that those who used own electric pumps (affordable irrigation) lost less than those who use the other types of pumps (hired electric pumps, hired diesel pump and own diesel pump) (Fig. 11). In appendix figure A2 and A3, we can see that this pattern holds regardless of canal access and district. We however find that, access to affordable irrigation will help substantially help reduce the yield losses in Banka, Lakhasirai and Madhepura.

**Fig. 12:**
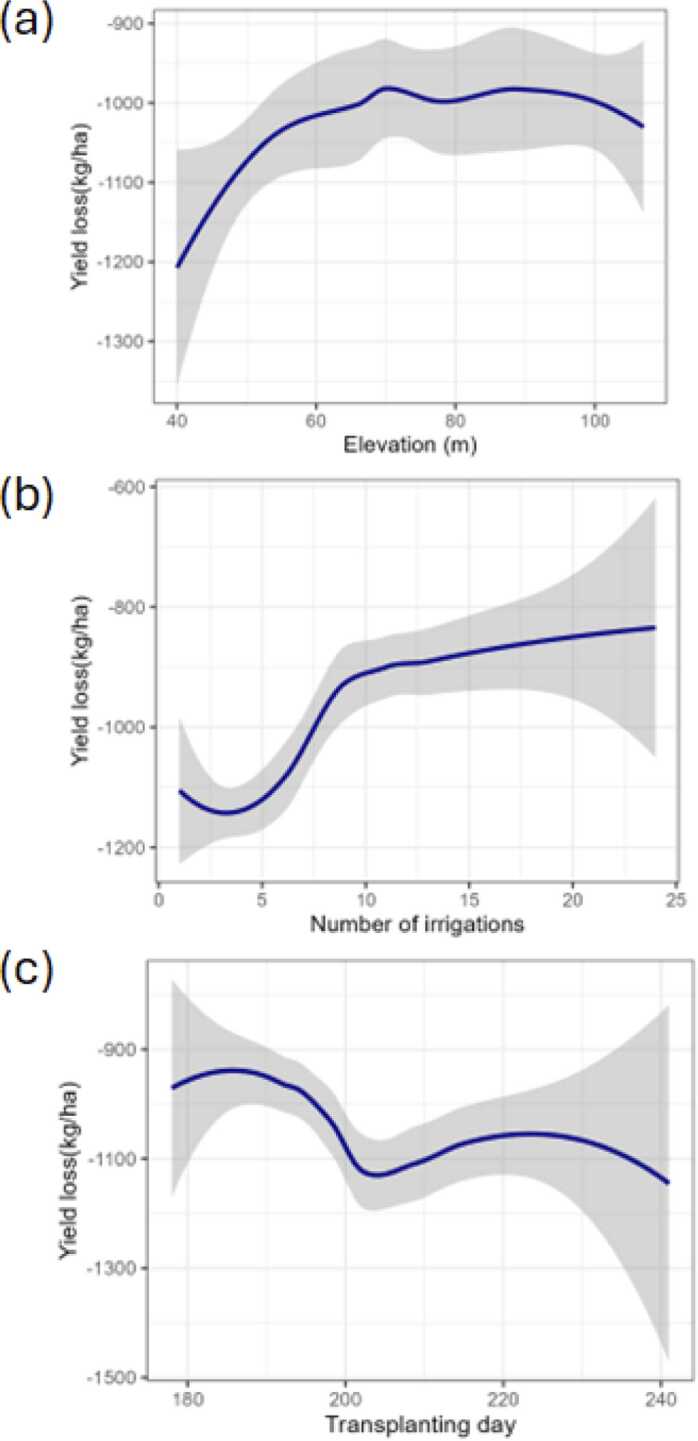
Effect of rice transplanting day, number of irrigations, and elevation on yield losses due to drought. Note: Panel (a) shows yield losses to drought differentiated by elevation. Panel (b) shows yield losses to drought differentiated by number of irrigations. Panel (c) shows yield losses to drought differentiated by transplanting day. The transplanting days can be interpretated as follows: 180th day is June 29th, 195th day is July 14th, 200th day is July 19th and 210th day is July 29th.

**Figure A4:**
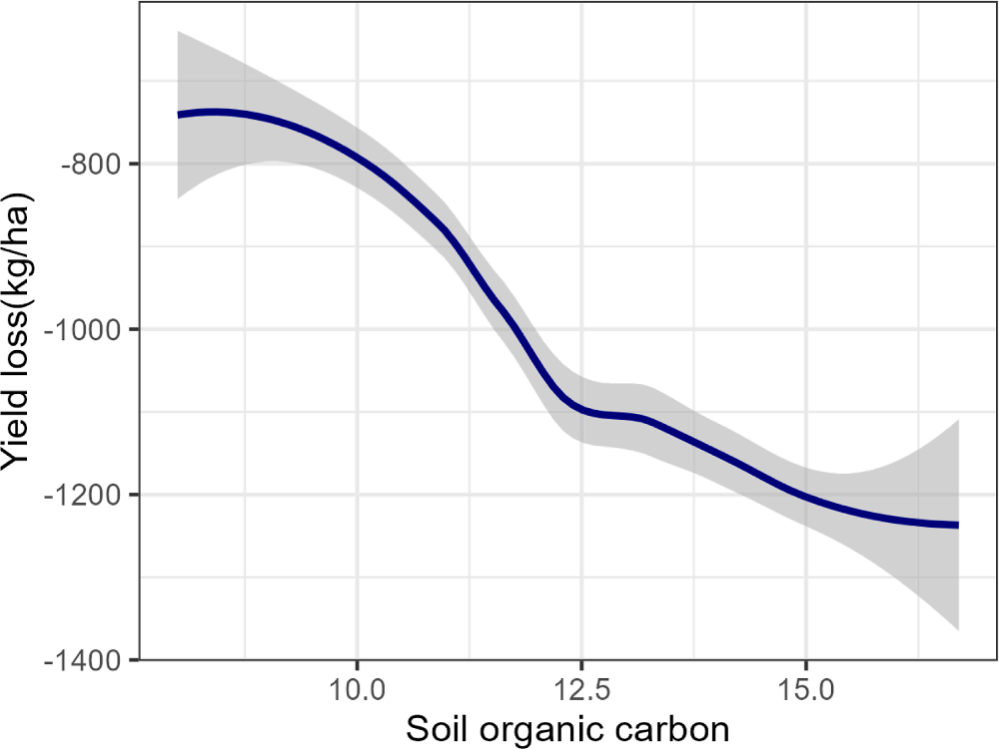
Yield loss due to drought across soil organic carbon gradient

**Figure A5:**
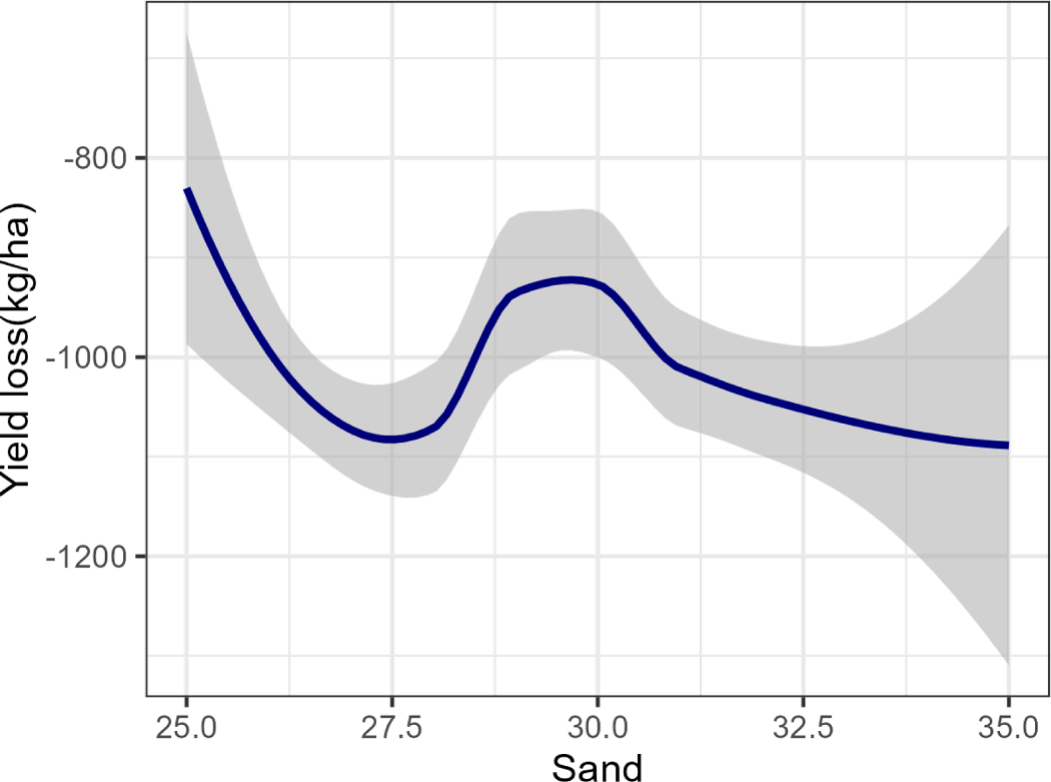
Yield loss due to drought across soil sand gradient

**Figure A6:**
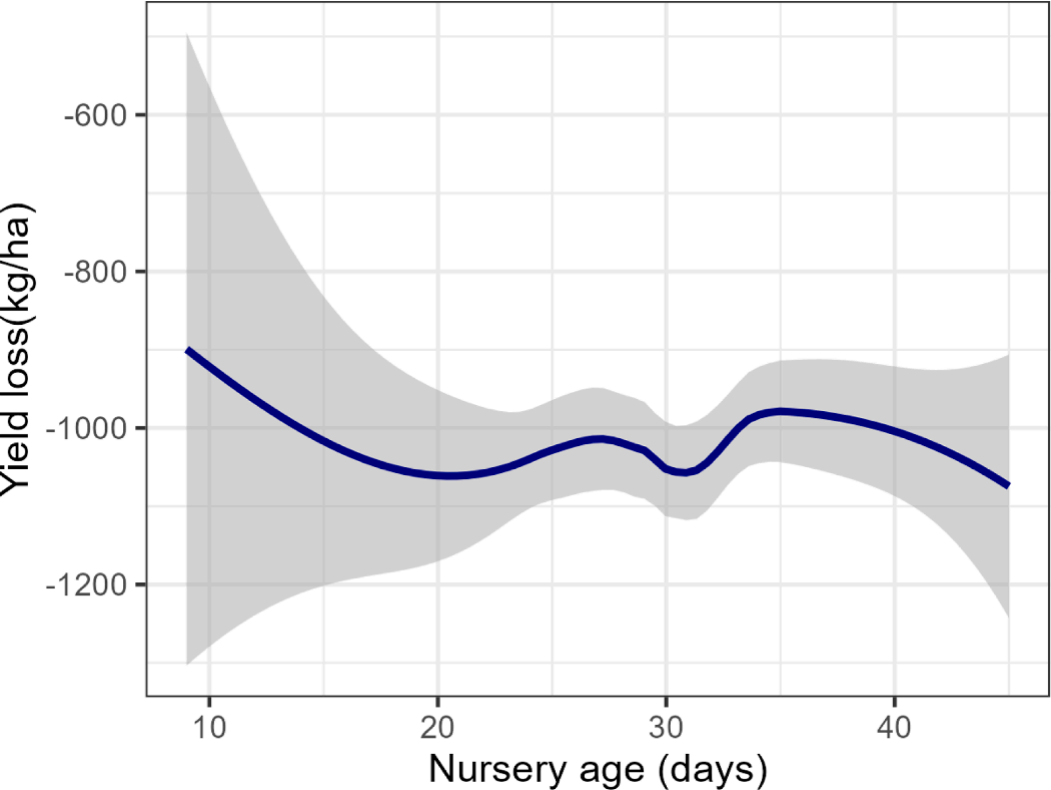
Yield loss due to drought across nursery age (days)

In addition to irrigation type, we can also check on whether these yield losses differ by elevation, number of irrigations applied, delays in transplanting, soil carbon, soil sand and nursery age (see appendix figures A4-A6 for the later three variables). Fig. 12a shows that low elevation areas are likely to have higher yield losses due to drought than high elevation areas but that the difference is very small. Fig. 12b shows that irrigating more than 5–10 times allows farmers to have substantially lower yield losses to drought. Fig. 12c shows that sowing after 195th day (July 14th) may lead to higher yield losses due to a drought. In addition to the irrigation type, those who manage to sow between June 29th and July 15th were likely to experience less yield losses due to drought in 2022.

## 4 Discussion

### 4.1 Farmer behavioural responses to droughts

Droughts reduce crop yields through the direct impacts on crop growth and through the behavioural responses that the farmers undertake in response to the shock. These responses can be counterproductive and may lead to further losses. This is because drought puts production of rice at risk (Bowden et al., 2025). Farmers without electric tubewell ownership might have expected large yield reduction due to drought. We argue that having access to affordable irrigation may help resolve some of these behavioural responses while also directly impacting the yields. However, farmers may stick to unfavourable practices which end up being worsened when there is a drought like condition.

We show that those with affordable irrigation are less likely to reduce their rice acreage (by among other strategies, leaving it fallow) and more likely to transplant on time, irrigate on time, and apply more irrigations. On crop area reduction, our results are consistent with Cui (2020) and Boyer et al. (2023) who using evidence from the United States argued that crop abandonment is a key farmer’s response to weather shocks.

Despite having electric tubewells, farmers in Bihar still wait for the monsoon instead of transplanting early. These farmers even wait longer than those without affordable irrigation. The key mechanisms through which affordable irrigation can affect yields is through optimal timing and volume of water applied to the crop. We did not collect information on the timing of the irrigation in our survey but recognize that this is very important just as the amount. And as reported in the survey, only 75 % of the farmers were able to irrigate when they needed to for those who reported a drought as compared to 98 % for those who did not experience a drought. On the number of irrigations, we find that those who experienced a drought applied a greater number of irrigations. This higher effect is more pronounced for those with access to own electric pumps. In terms of the hours per irrigation event however, we find that while this increased as well, farmers with own electric pumps used less hours per event. This is expected because electric pumps disperse more water per hour than diesel pumps.

Our survey evidence further supports this interpretation: among farmers who perceived themselves as transplanting late, 93 % reported that they waited for rainfall before proceeding. This pattern suggests that behavioural inertia and cognitive dependence on the monsoon continue to shape transplanting decisions, even among those with access to more affordable irrigation from electric tubewells. Such practices may reflect risk perceptions rooted in long-standing reliance on monsoon onset as a key signal for crop establishment. Therefore, while affordable irrigation relaxes cost constraints, complementary extension and information strategies are essential to encourage timely transplanting and to translate investment in electrification of irrigation into effective drought adaptation.

Complex weed flora and high population of weeds in drought year creates inequality in favor of weeds. The impact of droughts on weed pressure and the associated weed management decisions is complex and dynamic (Ramesh et al., 2017). It depends on which types of weeds are important for the crop and how these weeds respond to the climatic and canopy conditions.

### 4.2 Affordable irrigation effectiveness at moderating drought impacts

Affordable irrigation is considered the “master” variable for adapting to droughts in that it can incentivise yield improving agronomic behavioural responses while abating direct yield losses from water stress. However, some evidence from Bihar has cast doubt on subsidizing diesel pumps as it has been ineffective at reducing yield losses from drought (e.g., Kishore et al., 2015). This is contrary to optimistic studies that use historical drought events across India and consider all forms of irrigation as compared to rainfed agriculture. For example, Birthal et al. (2015) analyzed the impact of droughts on rice yields in India and found significant and increased abatement potential of irrigation (though decreasing in areas where groundwater is not available). Similarly, Amale et al. (2023) using the ICRISAT district level data reported a yield loss of only 155 kg/ha (6 %) due to delayed monsoon by about 20 days. Recently, Bowden et al. (2023) used the ICRISAT data and a random forest modelling approach to show that increase in area under irrigation helps in adapting to the impact of monsoon variability.

Access to affordable irrigation is expanding in Bihar with rapid adoption of electric pumps and high subsidy on electricity use for irrigation. Cheap irrigation can lead to groundwater overextraction in Bihar, especially given the increasing frequency of droughts. Rationalizing electricity tariffs for irrigation is essential to discourage unsustainable use of energy and groundwater. Coupling agronomic response effects and environmental thresholds, as in Urfels et al. (2024), can help in targeting irrigation infrastructure to areas where irrigation has the highest agronomic impacts and can be used sustainably.

### 4.3 Study limitations and further research

There are several limitations to our study. First, our study is based on cross-sectional data which limits the ability to conduct an ideal identification strategy for assessing the impacts of affordable irrigation on yields in drought years. In addition, the causal analysis of affordable irrigation requires causal moderation effects analysis (see, Bearth and Lechner, 2025) but this is beyond the scope of this paper as our interest is on just describing the moderating effects of affordable irrigation. Second, while affordable irrigation is a key adaptation strategy, there are increasing concerns of sustainability of this strategy given the potential depletion of groundwater with the increasing frequency of droughts. Our study did not consider these sustainability concerns.

Third, there are many other adaptation strategies including crop switching, migration, and focusing on non-farm activities that we do not capture in our study but potentially affect farmers’ ability to work on their farms and ultimately to yields. In addition, the behavioural agronomic responses we examine in this paper focus on the rice season, but we are aware that changes in the operations in the rice season also affect the wheat rabi season. Our further research will focus on a system analysis by looking at how drought affects the timing of paddy harvests and sowing of wheat. This will help in understanding the farmers’ behavioural agronomic responses in the subsequent wheat season.

Fourth, there is substantial attrition between the face-to-face survey and two follow-up telephone surveys. The potential bias was assessed by comparing the characteristics of the households. The characteristics of those who picked the telephone are largely similar to those who did not respond to the telephone interview. In addition, we used telephone data for the yield model while the bulk of the analysis was done using the full sample.

## 5 Conclusion

This paper has examined the impact of drought on agronomic behavioural responses and yields as well as how adaptive capacity through affordable irrigation helps in making better agronomic decisions. We find that drought substantially reduces yields through its impacts on the crop management decisions which are altered to manage the drought conditions. It is on these crop management decisions that farmers can take adaptive decisions that would allow them to be resilient to drought impacts. We find that droughts alter farmers management practices especially by reducing cropped area, increasing fallows, delaying transplanting, increasing number of irrigations, and weeding less. With affordable irrigation, farmers can make better decisions and use different inputs well except on the decision to transplant early. We found substantial yield losses to the 2022 drought averaging 0.94 t/ha (about 23 %) with considerable but not enough adaptation (0.3 t/ha) through affordable irrigation. We suggest that the massive rollout of affordable irrigation infrastructure that is underway in Bihar should be complemented with agronomic advisory services that help farmers implement drought mitigation strategies and promote judicious water use.

## Funding declarations

The study was funded by 10.13039/100000865Bill and Melinda Gates Foundation through the Cereal Systems Initiative for South Asia project ID: INV-029117.

## CRediT authorship contribution statement

**Sonam Sherpa:** Writing – review & editing, Supervision, Resources, Project administration, Methodology, Investigation, Funding acquisition, Conceptualization. **Prakashan Veetil:** Writing – review & editing, Writing – original draft, Validation, Supervision, Resources, Project administration, Methodology, Investigation, Funding acquisition, Conceptualization. **Avinash Kishore:** Writing – review & editing, Writing – original draft, Validation, Supervision, Resources, Project administration, Methodology, Investigation, Funding acquisition, Formal analysis, Data curation, Conceptualization. **Maxwell Mkondiwa:** Writing – review & editing, Writing – original draft, Visualization, Validation, Supervision, Software, Resources, Project administration, Methodology, Investigation, Funding acquisition, Formal analysis, Data curation, Conceptualization. **Andrew McDonald:** Writing – review & editing, Writing – original draft, Validation, Resources, Project administration, Funding acquisition. **Shishpal Poonia:** Writing – review & editing, Validation, Supervision, Project administration, Investigation. **Anton Urfels:** Writing – review & editing, Supervision, Methodology, Conceptualization. **Bhavani Pinjarla:** Writing – review & editing, Software, Data curation. **Satyam Saxena:** Writing – review & editing, Supervision, Project administration, Data curation. **Ram Malik:** Writing – review & editing, Supervision, Resources, Project administration, Funding acquisition. **Peter Craufurd:** Writing – review & editing, Supervision, Resources, Project administration, Funding acquisition. **Anurag Ajay:** Writing – review & editing, Supervision, Project administration, Data curation.

## Declaration of Competing Interest

The authors declare that they have no known competing financial interests or personal relationships that could have appeared to influence the work reported in this paper.

## Figures and Tables

**Table A1:** Summary statistics of full list of variables by reported drought incidence, Bihar 2022

	Drought (344)		No drought (168)		Total	
Variables	N	Mean	N	Mean	N	Mean
**(1.) Land use**						
Total operational landholding (acres)	344	4.25	168	5.89	512	5.07
Rice cultivation area in 2022 (acres)	344	2.60	168	5.75	512	4.17
Rice cultivation area in 2021 (acres)	344	3.22	168	5.83	512	4.52
Fallow land (acres)	344	1.32	168	0.03	512	0.68
Share of fallow land (Fallow land_acres/ Total operation land acres)	344	0.29	168	0.01	512	0.15
**At least one plot in fallow in Kharif 2022**						
*Yes*	344	0.76	168	0.28	512	0.52
**Reasons for fallow land**						
*Poor labor availability*	262	0.07	48	0.00	310	0.03
*The plot does not have access to irrigation*	262	0.08	48	0.04	310	0.06
*The plot is waterlogged*	262	0.11	48	0.00	310	0.05
*Was waiting for rain*	262	0.74	48	0.94	310	0.84
*Others*	262	0.00	48	0.02	310	0.01
**Type of land ownership**						
*Owned*	344	0.85	168	0.93	512	0.89
**Drainage class of the land**						
*Low land*	344	0.19	168	0.18	512	0.18
*Medium land*	344	0.74	168	0.77	512	0.76
*Up land*	344	0.07	168	0.04	512	0.06
*Very low land*	344	0.00	168	0.01	512	0.00
**(2.) Irrigation**						
**Type of pump used for irrigation purposes**						
*Hired diesel pump*	344	0.30	168	0.14	512	0.22
*Hired electric pump*	344	0.19	168	0.05	512	0.12
*Others*	344	0.00	168	0.21	512	0.11
*Own diesel pump*	344	0.19	168	0.15	512	0.17
*Own electric pump*	344	0.31	168	0.44	512	0.38
**Sources of Irrigation**						
*Ahar pyne or gabra*	344	0.02	168	0.00	512	0.01
*Canal*	344	0.06	168	0.81	512	0.44
*Dugwell*	344	0.01	168	0.00	512	0.00
*others*	344	0.01	168	0.00	512	0.01
*Public tubewell*	344	0.14	168	0.01	512	0.07
*Tubewell*	344	0.76	168	0.18	512	0.47
**Irrigation when needed**						
*Yes*	344	0.75	168	0.98	512	0.86
**Main reasons for the delay of irrigation**						
*High cost of irrigation*	87	0.43	4	0.25	91	0.34
*Water table too deep*	87	0.32	4	0.00	91	0.16
**(3.) Crop Establishment**						
**Crop establishment method**						
*Manual transplantation*	344	1.00	168	1.00	512	1.00
Age of seedlings when transplanting (days)	343	30.05	168	31.14	511	30.60
**Perception on lateness of transplanting**						
*Early*	344	0.01	168	0.02	512	0.01
*Late*	344	0.49	168	0.31	512	0.40
*On time*	344	0.41	168	0.67	512	0.54
*Very late*	344	0.10	168	0.00	512	0.05
**Reason of crop establishment delay**						
*I did not have the money to pay for the inputs*	201	0.00	52	0.02	253	0.01
*Labor was not available on time*	201	0.04	52	0.15	253	0.09
*Rain was late, I was waiting for the rains*	201	0.93	52	0.56	253	0.74
*Water was released late in the canals*	201	0.04	52	0.27	253	0.16
**(4.) Weed management**						
**Type of weed management applied**						
*Both spray and manual weeding*	344	0.09	168	0.52	512	0.31
*Only manual weeding*	344	0.75	168	0.38	512	0.56
*Only sprayed herbicides*	344	0.16	168	0.10	512	0.13
Manual weeding times	289	1.27	151	1.21	440	1.24
Herbicide spray times	85	1.07	105	1.11	190	1.09

**Figure A1:**
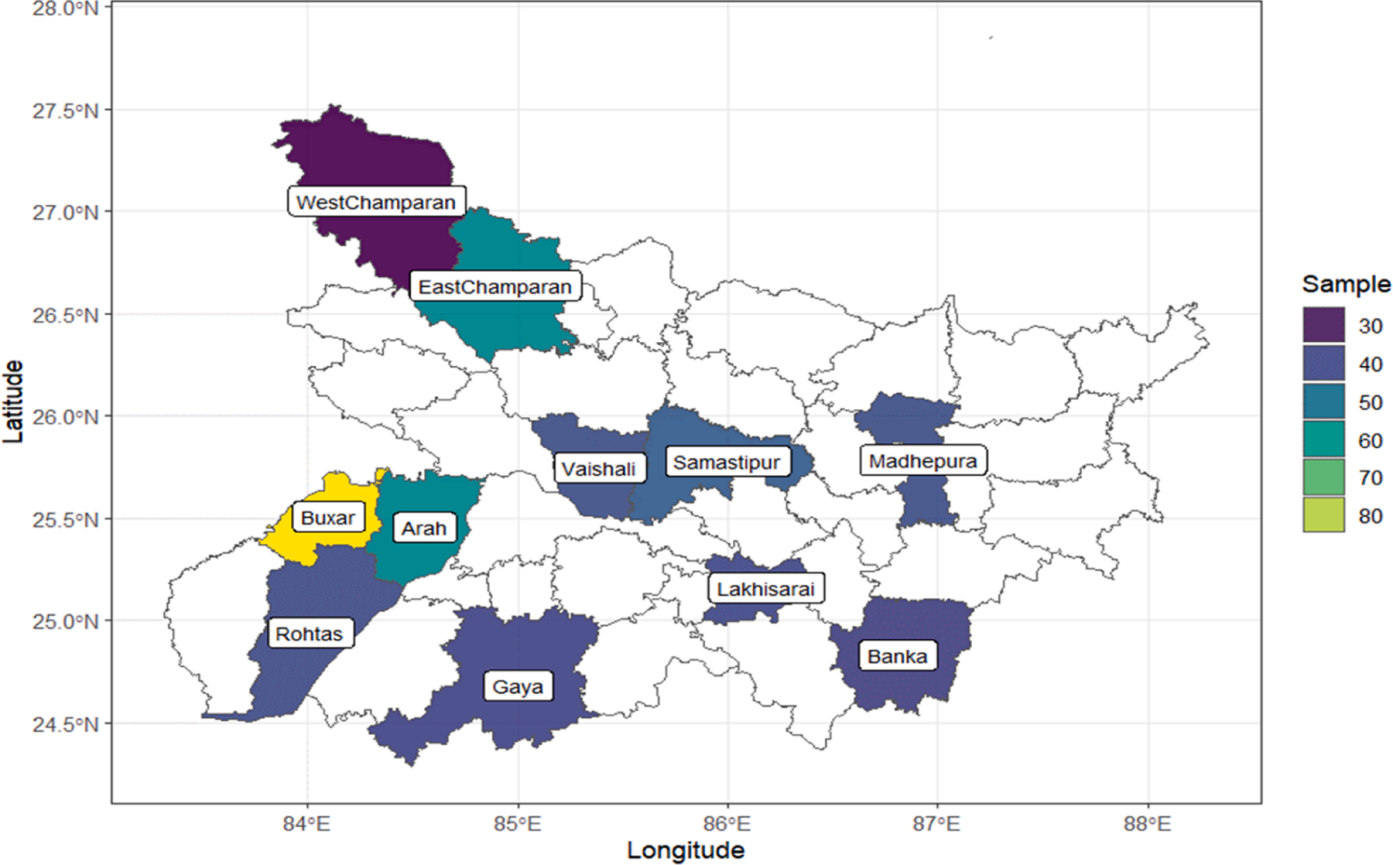
Sample size across the selected districts

**Figure B1:**
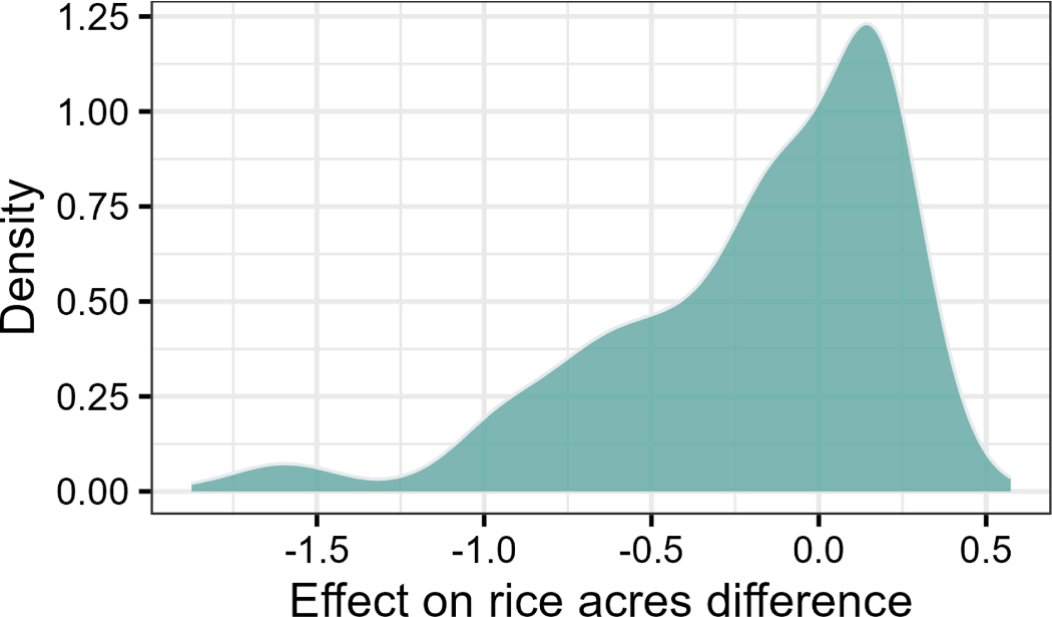
Distribution of conditional average treatment effects of farmer reported drought on rice acreage

**Figure B2:**
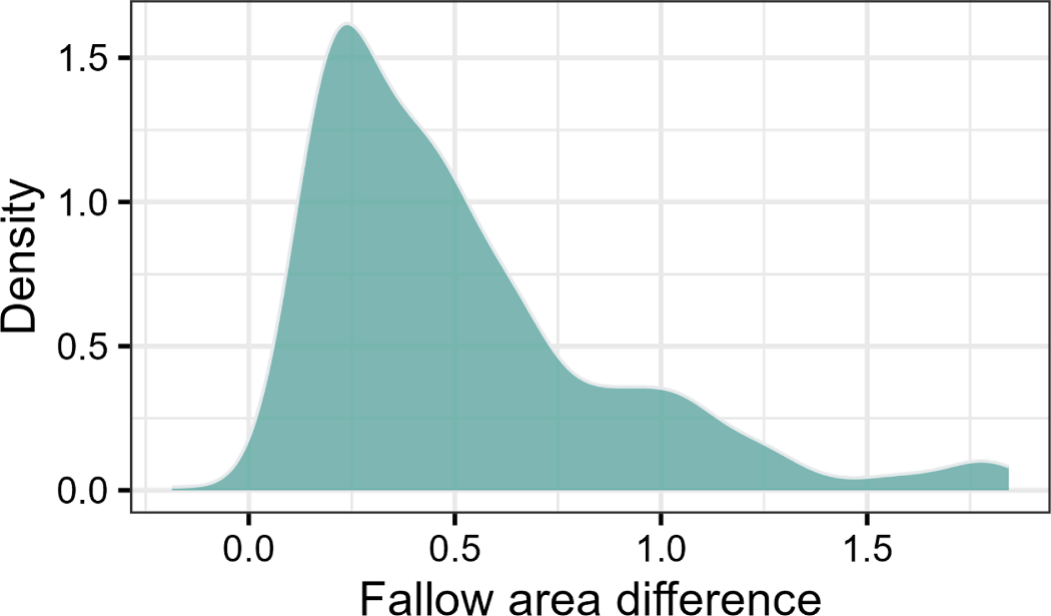
Distribution of conditional average treatment effects of farmer reported drought on fallow acreage

**Figure B3:**
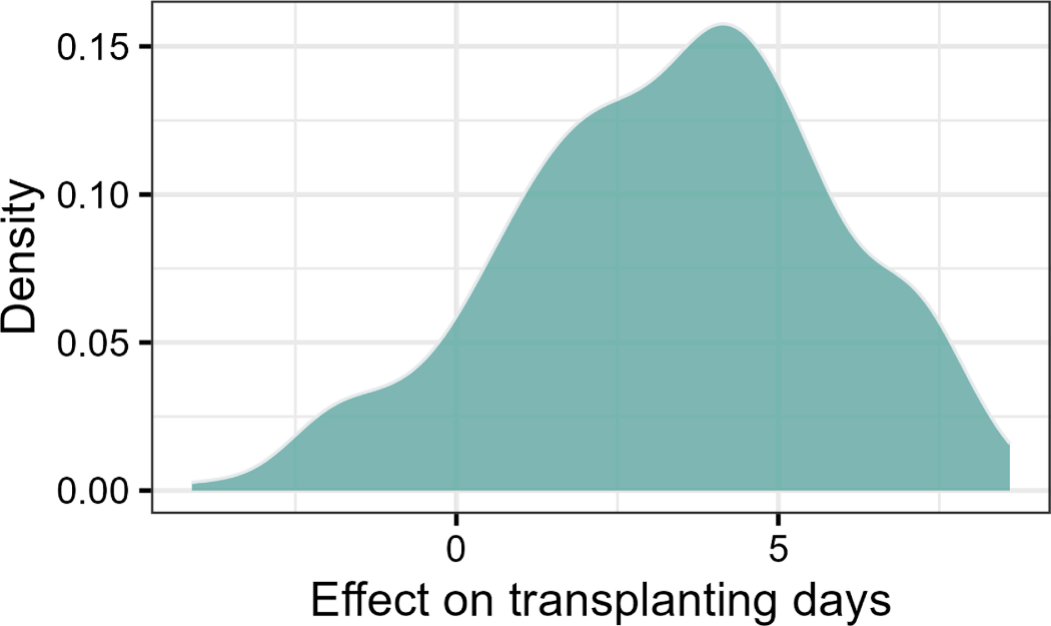
Distribution of conditional average treatment effects of farmer reported drought on rice transplanting day

**Figure B4:**
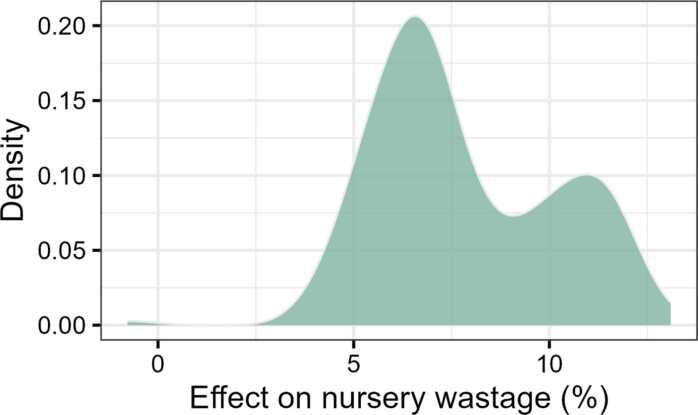
Distribution of conditional average treatment effects of farmer reported drought on rice nursery wastage

**Figure B5:**
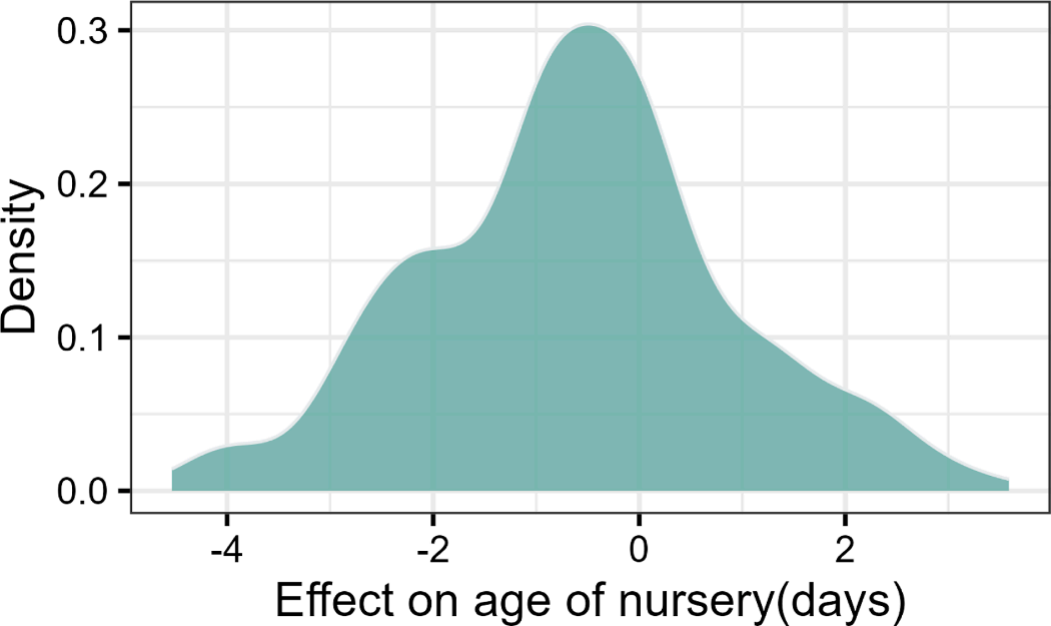
Distribution of conditional average treatment effects of farmer reported drought on age of nursery (in days)

**Figure B6:**
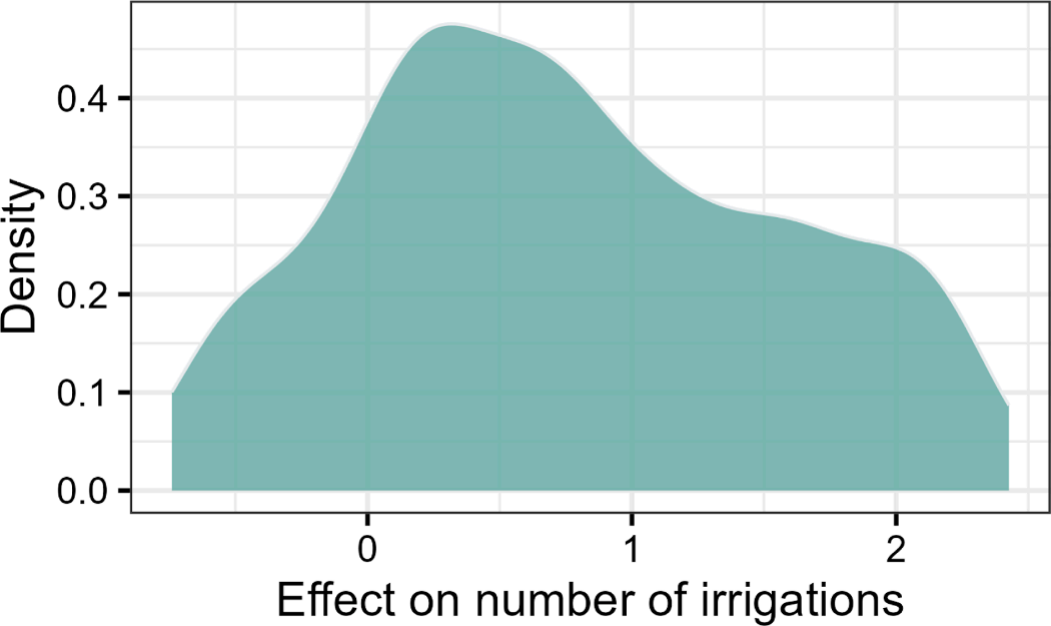
Distribution of conditional average treatment effects of farmer reported drought on number of irrigations

**Figure B7:**
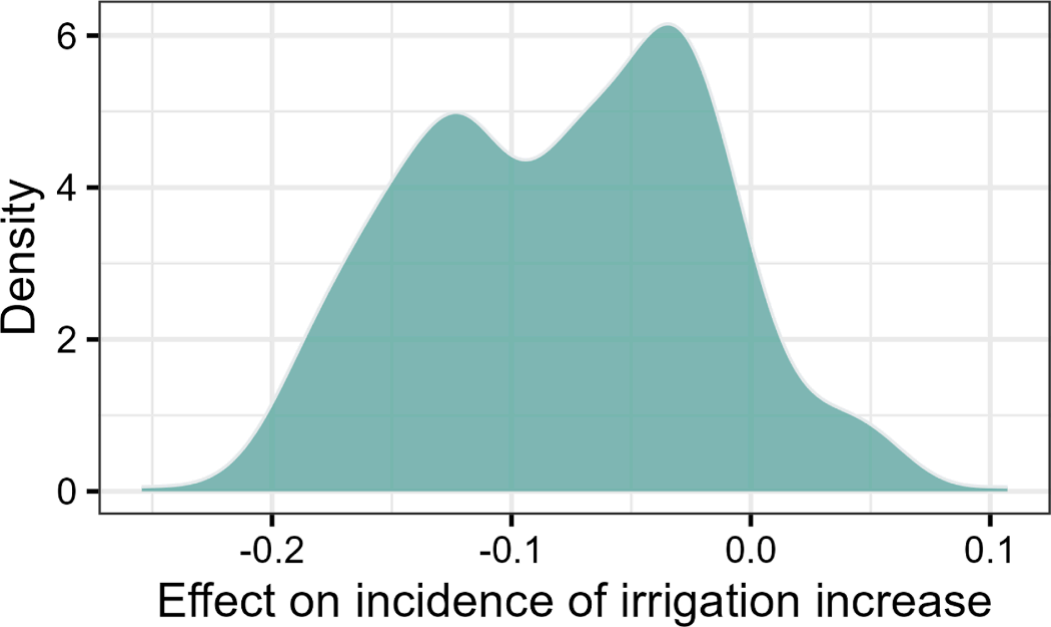
Distribution of conditional average treatment effects of farmer reported drought on incidence of irrigation increase

**Figure B8:**
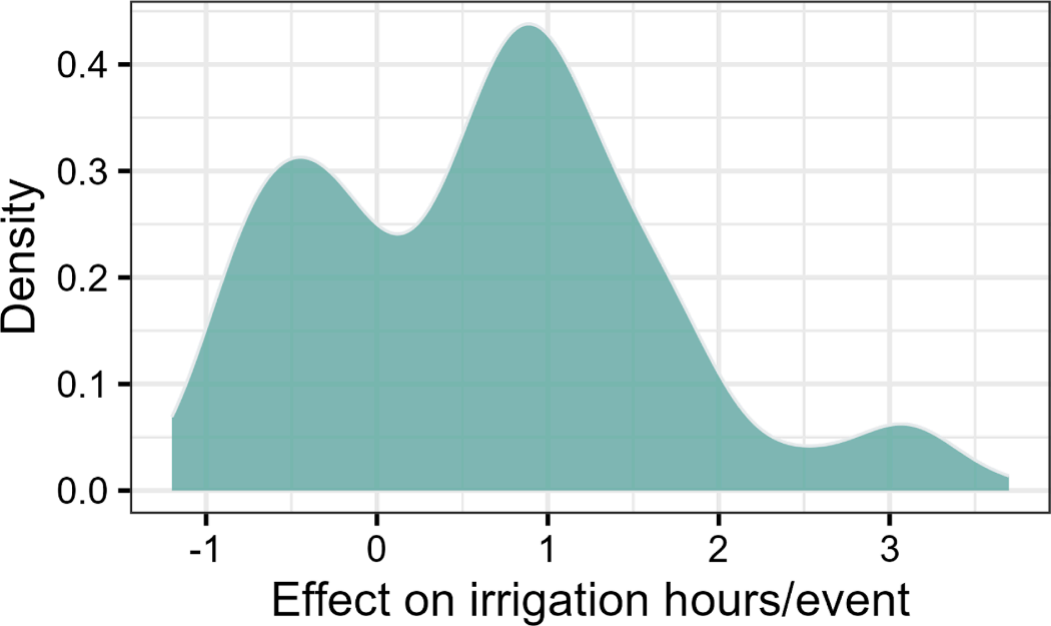
Distribution of conditional average treatment effects of farmer reported drought on irrigation hours per irrigation event

**Figure B9:**
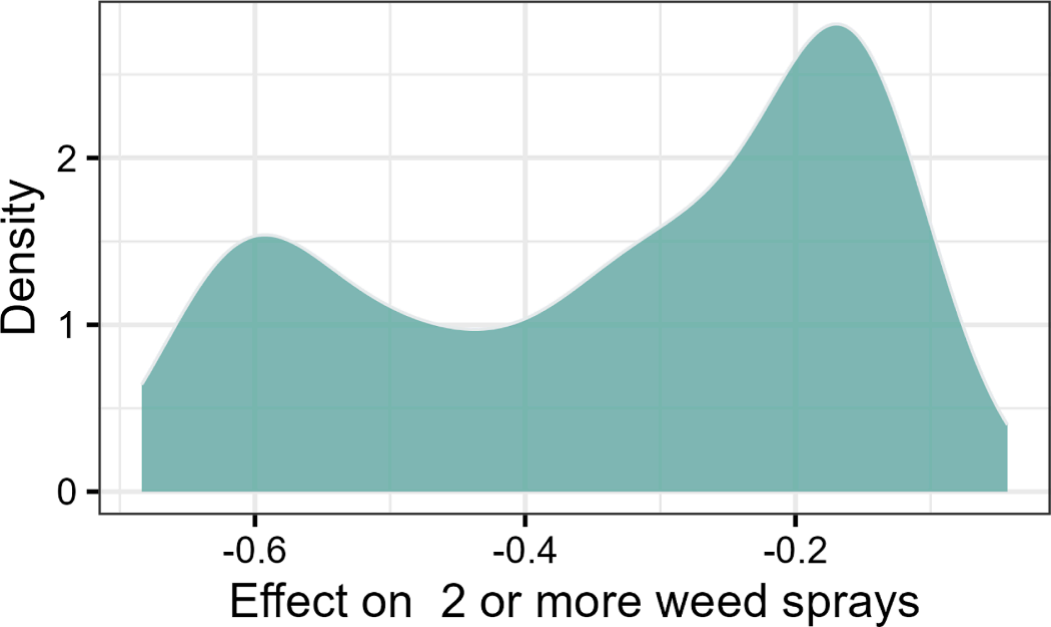
Distribution of conditional average treatment effects of farmer reported drought on two or more weed sprays

**Figure B10:**
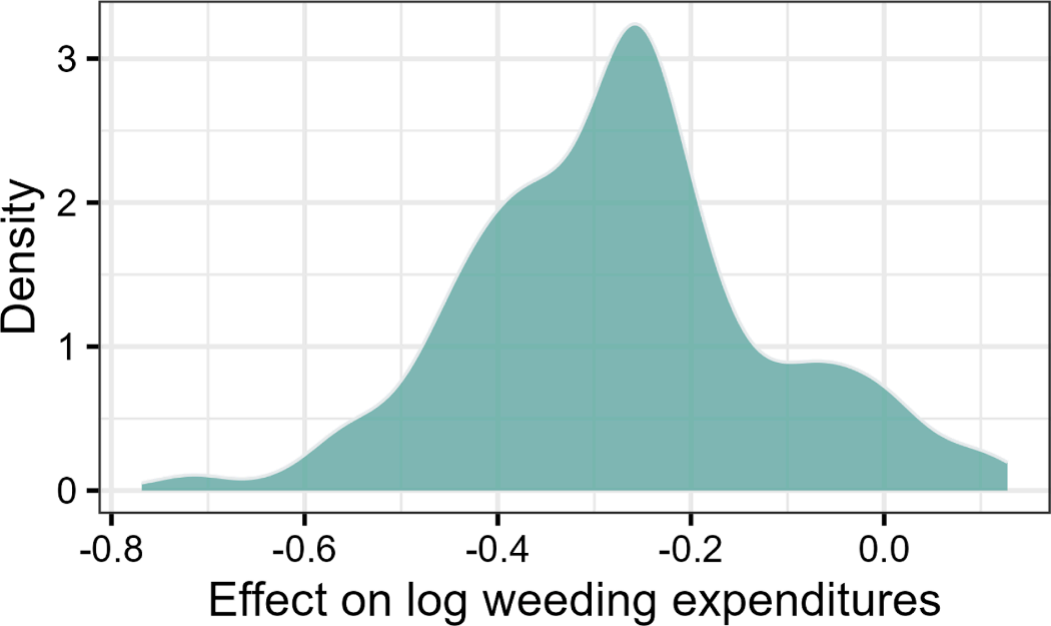
Distribution of conditional average treatment effects of farmer reported drought on log weeding expenditures

## Data Availability

Data will be made available on request.

## Footnotes

1 https://indianexpress.com/article/india/75-pc-of-average-area-in-up-bihar-jharkhand-see-deficit-rainfall-imd-8181392/

2 According to Crop Weather Watch Group (CWWG) meeting on 16th September 2022 (https://agriwelfare.gov.in/sites/default/files/CWWG%20Key%20Points%20as%20on%2016092022_compressed%20%281%29_0.pdf), a group within the Directorate of Economics and Statistics, Department of Agriculture and Farmers Welfare.

3 Because, for example, farmers may be changing fertilizer practices or other things that aren’t reflected in regression models (i.e., omitted variables).

4 We do not implement this in this paper because our interest is on the causal drought impacts and associations (not causal) to affordable irrigation. The reader is referred to Bearth and Lechner (2025) for a causal moderation effects analysis.

5 Ideally, one would need multiple years of data rather than a unique previous year. We do not have outcome variables at household level except for these two years.

6 Cerqua et al. (2023) describes this problem in causal inference.

7 To avoid confusing the reader with the inconsistent estimates from the conventional estimators, we do not report these in this paper but are available from the authors upon request.

8 We estimate the generalized random forest model using *grf* R package.

9 The geographical control variables (population density, elevation, soil sand, and soil carbon) were extracted for each farm geocoordinate from *geodata* R package.

10 We use SPI instead of just rainfall because by indexing against the mean rainfall, SPI quantifies how much precipitation deviates from the long-term average for a particular location and period. SPI offers a more comprehensive and standardized approach to drought assessment, considering temporal distribution, standardization, flexibility, and probabilistic analysis. Mean rainfall, while informative, may not capture the variability and persistence of drought conditions.

11 We recognize that there are many drivers of NDVI difference and that putting a hard threshold close to zero may not be ideal, but our goal is just to check if there is a general concordance with the self-reported drought.

12 The yield results should be interpreted with caution because there was substantial attrition because the yield variable was collected using a follow-up telephone survey.
